# Dental Education

**Published:** 1880-07

**Authors:** W. H. Morgan


// THE DENTAL REGISTER.

Vol. XXXIV.] JULY, 1880. [No. 7.

### DENTAL EDUCATION.

BY W. H. MORGAN, D. D. S.

*Read before the Tennessee State Dental Society.*

Mr. President and Gentlemen of the Tennessee Dental Association:—The duty of preparing a paper on dental education for this occasion has been assigned me by your executive committee. This duty I have in my humble way attempted to perform in the following, which, if you will bear with me, I will proceed to read. And while we meet here on this glad centennial occasion, it may be well to remember that it is not in any sense the centennial of dentistry here in Tennessee or indeed in America. One hundred year ago, so far as history records or tradition relates, there was not a dentist on the American Continent, and that it was not until four years after, that is in 1784, that we have any account of any one engaged in the practice of dentistry in this country. It is true that it is fair to infer that the elder Greenwood, in Boston, and Baker, in Philadelphia, did practice dentistry before that period, but we have no positive record or tradition to establish the fact. So that the position of dentistry as a science and art is the outgrowth of less than a century, a mere point of time in the history and probable duration of our race.

This subject is to-day by far the most important one before our profession, both in its present relations and bearings and its future influence on our standing and destiny as a separate, learned and liberal profession, especially so as to other professions and most especially toward our mother profession, medicine.

It might be profitable here to take a glance at a part of what has been done in this comparatively short time. And in considering the subject to go back and review somewhat in detail scraps of our history and canvass the views and hopes of some of those who have gone before us, who have given shape to our present modes and plans for the education of those who propose to enter our ranks and who "builded better than they thought."

In the year 1784 two dentists made their appearance on the arena, and then they seem to multiply in accordance with or obedience to the demand for their services, practicing their profession and imparting a knowledge thereof as a secret art, and receiving for the latter, as I shall show you, very large fees. This continued down to 1840, when the American Society of Dental Surgeons was organized. In 1842 the Virginia Society of Dental Surgeons was organized and obtained a charter from that State, which enabled them to legally confer degrees, the degree of Fellow of the Virginia Society of Dental Surgeons. This date constitutes the first epoch in dental education in America, the organization of these associations and of the Baltimore College of Dental Surgery, in 1840, was literally the beginning of our status as a profession, and was the womb out of which was born all that is worthy and liberal and lofty in sentiment in our profession. I am persuaded that these early efforts of associations were even less appreciated than like associations at the present day, although their large number would indicate a great improvement in this direction. The truth is that these early associations are the germ out of which has grown, either directly or indirectly, all that to-day lifts us above the mere artizan or mechanic. Its first fruit was a journal devoted exclusively to our interests, the "Baltimore Journal and Library of Dental Science," still in existence as the "Baltimore Journal of Dental Science," and although there had appeared before this a few books on dentistry in this country, this was the beginning of popular dental literature, was the work of the American Society of Dental Surgeons, and was its property. This was the first epoch in dentistry, and for the next forty years we have been advancing with speed, ever accelerating speed, toward the goal of our ambition—perfection in dental science. And though to-day we may be, and as I believe are, a long way off, we are progressing in the right direction, and hope and faith in the future is bright. Within less than 40 years there have been more than 75 dental associations formed, about 70 of which are now in existence and in working order, many of which meet monthly and all at least once a year. Can any one estimate the amount of information which is diffused and gathered by all this effort; can they ever approximate their value in education and advancement in knowledge of those who attend upon their proceedings. Not only their value as educational institutions in the ordinary acceptation of that term the impartation of knowledge is to be considered, but their influence from another and possibly more important stand point, that is the cultivation of a proper spirit; the cultivation of that liberality and brotherhood between members near each other, and that *esprit du corps* that makes a few invincible in the accomplishment of a given purpose. Within the same period 20 dental colleges have been organized; of this number 14 are still in existence. Up to 1876 these colleges had regularly graduated 2,865 students, and I have seen it stated recently that since that date, about 850 more had been graduated, making in all about 3715 regular graduates, men who had complied with the requisitions of these schools and passed their examinations. In the early history of our profession the class of dentists capable of giving instruction was necessarily very small, and many of this small number disinclined for want of time and other reasons to do so. Such was the value of a certificate from a prominent dentist that large fees were demanded. Five hundred dollars, says L. S. Parmley, was commonly asked a a pupil for office instruction, and in those days $500 was equal to $2,000 now in its buying capacity. In lectures on "The Natural History of the Teeth," by L. S. Parmley, London, 1820, page 90, you will find this extraordinary passage: "Mr. Parmley being desirous that his peculiar treatment of the teeth, his operations and general views on the subject should become as widely diffused as possible for the common benefit of society, undertakes to qualify gentlemen of liberal education for practice as dentists on the following terms: For practice in London, $1,000; for any other city in England or America, $700: for foreign practice, $500." This last sentence, says a recent writer, must not be regarded as savouring of quackery. So far as advertising is concerned, the dental ethics of that day did not exclude advertising as unprofessional. My old partner and friend, Dr. T. B. Hamlin, informed me that he paid one teacher after he had been in practice several years, $900 for three months instruction in operative dentistry; this must have been as late as 1832 to 1835. At this period it was exceedingly difficult to get books or any kind of literature on the subject, and this continued until the inauguration of the Baltimore college in 1840. My first teacher informed me when he solicited and prevailed on me to study dentistry, that there were two books on the subject and he had both of them and that they should be at my service. You will no doubt be astonished at his ignorance, but he was not many years behind, and the rule was in that day that the more ignorant he was the more students he was likely to have, because his want of knowledge made the attainment of a certificate or endorsement of qualification easy to obtain, and I regret to say that this general rule holds good in the present day. Although many doubts were entertained of the plan and enterprise of dental colleges at the time of their first organization, 1840–44, many good men calling in question the propriety of their establishment, these prejudices soon gave way before an enlightened public opinion, so that in a few years only a few old fogies, or those of most limited knowledge, called in question their wonderful usefulness in qualifying men for an intelligent and skillful discharge of their duties as dentists. True, many entered and continue to enter the profession, without a collegiate course, mainly because the public do not positively demand such a course of training, and because the want of means and the fact that their tutors did not take such instruction furnishes a ready excuse not to do so; but as I know that my hearers on this occasion do not sympathize with such frivolous excuses, I will proceed to another branch of this subject.

About the year 1850 a controversy arose as to the best mode of organizing a dental school. Drs. Gardette, of Philadelphia, C. C. Allen, of New York, and B. Wood, of this city, taking the ground that one chair in a medical college was all that was needed, in addition to the medical teaching had in such an institution, to qualify men for the practice, but they were so overwhelmed by Harris, Wescott, Cone and others, that solemn silence reigned over this proposition for many long years here and there. I can only call to mind two respectable medical colleges who adopted lectureships on dentistry, and both these have long since abandoned them. The most talented and cultivated gentleman whom I have known to fill this anomalous lectureship informed me that the result was disastrous, that when the graduates from his school failed in general medicine (such colleges have given no diplomas except in medicine) they took up dentistry, so they had quite a number of half made dentists, to use his expression, in the region from which his school drew his patronage. The next move was to have students take the tickets of some regular medical college in anatomy, physiology, chemistry and materia medica, and have the strictly dental branches taught by dentists, and while I saw many serious objections to this plan, I favored and advocated it, believing it to be an advanced step over the then superficial (comparatively) course of instruction in the last named sciences. The discussion of this subject from 1850–54–56 had the most salutory influence upon the schools then in existence, and this evil was remedied by adding appropriate chairs in our dental colleges. I believe the Ohio College of Dental Surgery has the honor of giving the first full course in chemistry from its appropriate chair. When these evils were remedied I gave my adherence to the present system, that is of having full professorships for the medical branches and the like professorships for the dental branches. Medicine taught by medical men and dentistry by dental men, and only teaching so much of general medicine as the needs of the dentist demands, and so permitting the student to give the remainder of his time to his immediate calling. While I am in favor of a broad education for the dentist, there are many things in general medicine for which he has very little use; still, if he has the time and means I would have him earn his M. D., and then base his dental education upon the knowledge acquired. Years ago Dr. Thacksten, of

Va., said most truly our colleges, meaning dental colleges; must meet the demands of the age or go down and be succeeded by others in harmony with the times and measuring up to all the duties and responsibilities of advanced and advancing science. That great defects exist in our present system of teaching none will deny, but we are making some advance and I trust I shall see all the dental colleges in this country taking a decided step in advance in their announcements for the session of '80–'81.

Dr. Keely, of Ohio, in a report read in '74 or '75 said, "The requirements of dental practice are constantly enlarging, and can only be met by a deeper and more intelligent study of fundamental principles, and a more perfect adaptation of them in practice. The truths of the profoundest science must be embodied in the most intelligent and elaborate of art. Hence dental surgery, like any other profession, demands equal facility in abstract investigation and concrete adaptation." How are we to get all this? Dr. A. W. Freeman said of students, "They should have a good English education at least. Let them understand that they have entered upon no short cut or easy way to a livelihood." The American Dental Association, very democratic and very loose in its professional morals and practice, resolved, "That it is the belief of this society that no person should be admitted as a student of dentistry who is not fully qualified mentally, morally and physically, nor for a less term then three years." The American Dental Association resolved, "That no student should be graduated from any dental college without at least three years instruction." And further resolved, "That this Association recommends to all local societies the adoption of rules prohibiting their members from taking students for a less period than three years." You will bear in mind that this last eminates from the American Dental Association, the highest and most authoritative body of dentists in the world, the *senatus senatorum* of dental science, and to which belong many of the most learned dentists and distinguished scientists who speak our tongue. I might go on with such quotations as the above to establish the fact that the profession is looking to a higher education of those who seek to enter its portals; this fact alone demonstrates the necessity of such a demand, a demand for a more thorough education before they are permitted to bear from our colleges a diploma. If I do not mistake the signs of the times, the tendency in this and other countries is to the university system, a system founded upon the observation and experience of the learned world ; a school or system of schools in which a man can learn anything he may wish to know ; where the teaching is exactly adapted to the acquirements of the students, and not when all are thrown promiscuously together as in our medical and most of our dental colleges, and all the advanced and the ignorant listen to the same lectures, the same teaching.

I trust the day is not distant when the higher educational institutions of this country will all be conducted on this plan, and when the students will be advanced in exact proportion to their attainments without regard to time spent in college, and when a diploma will be evidence—full evidence that the possessor has won such a distinction by his or her acquirements, and not by the time spent in its pursuit. Diplomas most difficult to obtain I know are not most sought after, but they are most valued when obtained, and indicate most to the intelligent portion of the public. I know that in many localities graduation in dentistry has been made cheap and easy, but I also know that a day of reckoning will surely come, that truth will be vindicated and condign punishment will be meted out to the offending colleges which make graduation easy. A plan is now on foot to secure the co-operation of the four universities in this which have dental departments to raise their standard of scholarships and requirements for graduation. I hope to see it consummated.

